# Membrane Proteins in Action Monitored by pH-Responsive Liquid Crystal Biosensors

**DOI:** 10.1021/acsami.4c06614

**Published:** 2024-06-06

**Authors:** Peng Bao, Kyle Phillips, Rasmita Raval

**Affiliations:** Open Innovation Hub for Antimicrobial Surfaces, Surface Science Research Centre, Department of Chemistry, University of Liverpool, Liverpool L69 3BX, U.K.

**Keywords:** liquid crystal biosensor, pH sensor, membrane protein, bacteriorhodopsin, purple membrane

## Abstract

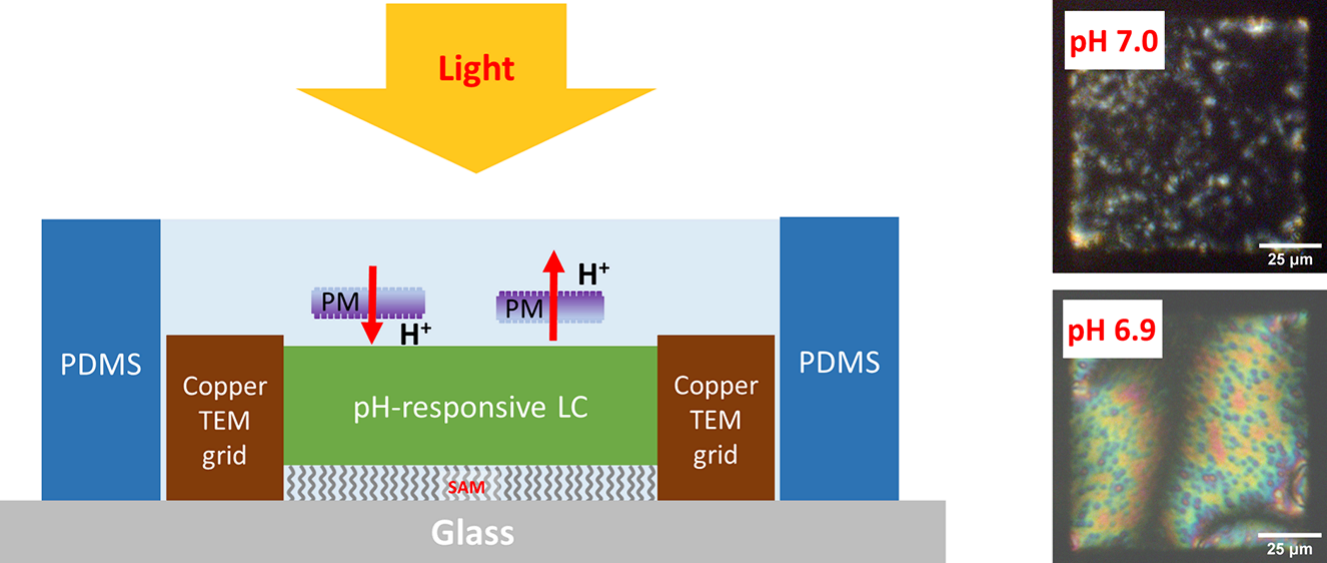

Liquid crystal (LC) biosensors have received significant attention for their potential applications for point-of-care devices due to their sensitivity, low cost, and easy read-out. They have been employed to detect a wide range of important biological molecules. However, detecting the function of membrane proteins has been extremely challenging due to the difficulty of integrating membrane proteins, lipid membranes, and LCs into one system. In this study, we addressed this challenge by monitoring the proton-pumping function of bacteriorhodopsin (bR) using a pH-sensitive LC thin film biosensor. To achieve this, we deposited purple membranes (PMs) containing a 2D crystal form of bRs onto an LC-aqueous interface. Under light, the PM patches changed the local pH at the LC-aqueous interface, causing a color change in the LC thin film that is observable through a polarizing microscope with crossed polarizers. These findings open up new opportunities to study the biofunctions of membrane proteins and their induced local environmental changes in a solution using LC biosensors.

## Introduction

Liquid crystal (LC) materials have been widely used in industry for LC displays. In the past 20 years, LC materials have gained much attention due to their potential application as biosensors for point-of-care devices.^1−7^ Two main forms of LC biosensors have been investigated, namely, those based on the interaction of LC with chemically modified solid interfaces and those based on the interaction of LC with water-soluble molecules at the LC-aqueous interface. The second type of biosensor is suitable for the study of biological molecules or cells. Many different kinds of biological molecules and entities have been detected using LC biosensors, including peptides, proteins, lipids, DNAs, enzymes, bacteria, and mammalian cells, but not membrane proteins.^8−15^ The main challenge for studying membrane proteins using LC biosensors is the integration of lipid bilayers with LC materials.

Membrane proteins are embedded in or attached to the lipid bilayer of cell membranes of bacteria or mammalian cells. They play a crucial role in a wide range of cellular processes, including cellular signaling, transport of molecules/ions through the membrane, maintaining the integration of the cell membrane, etc.^16^ In the human body, one-third of all proteins are membrane proteins, which are the target of more than 60% of modern drugs.^17^ The study of membrane proteins is challenging due to their instability when isolated from their native environment, though they have been intensively studied by X-ray crystallography, single-particle cryoelectron microscopy (cryo-EM), receptor–ligand binding assays, etc.^18^ However, to the best of our knowledge, LC biosensors have not been demonstrated for studying membrane proteins.

Most LC biosensors are based on the nematic phase of hydrophobic thermotropic LCs. Therefore, it is challenging to coat a lipid bilayer on the surface of an LC, although a lipid monolayer can be easily formed.^8−10,15,19,20^ For example, the Abbott group has pioneered research on lipid monolayer-coated LC biosensors.^9,15,19^ Peng et al. demonstrated the on-chip detection of the antimicrobial peptide SMP43 by combining microfluidics with lipid-coated LC droplets.^10^ Recently, there have been some attempts to integrate lipid bilayers with lyotropic LCs.^21,22^ In these studies, further integration of membrane proteins with the membrane–LC system has not been demonstrated.

To bypass the difficulty of integrating lipid bilayers with thermotropic LCs, we employed native purple membrane patches as a model system to show that their biofunction could be studied by LC biosensors. Purple membranes are two-dimensional crystalline arrays of bR molecules, normally found in the membrane of halophilic archaea, and are one of the most studied membrane proteins.^23,24^ BRs in the purple membrane can pump protons through the purple membrane patches from the cytoplasmic surface to the extracellular surface under the illumination of visible light (the absorption peak is around 570 nm).^25^ Therefore, by placing PMs at the LC-aqueous interface, we expect a change in the local pH, especially in the narrow space between the LC and purple membranes or in the proximity of the region. We then use a pH-sensitive LC biosensor to report the change in the local pH value.

**Figure 1:**
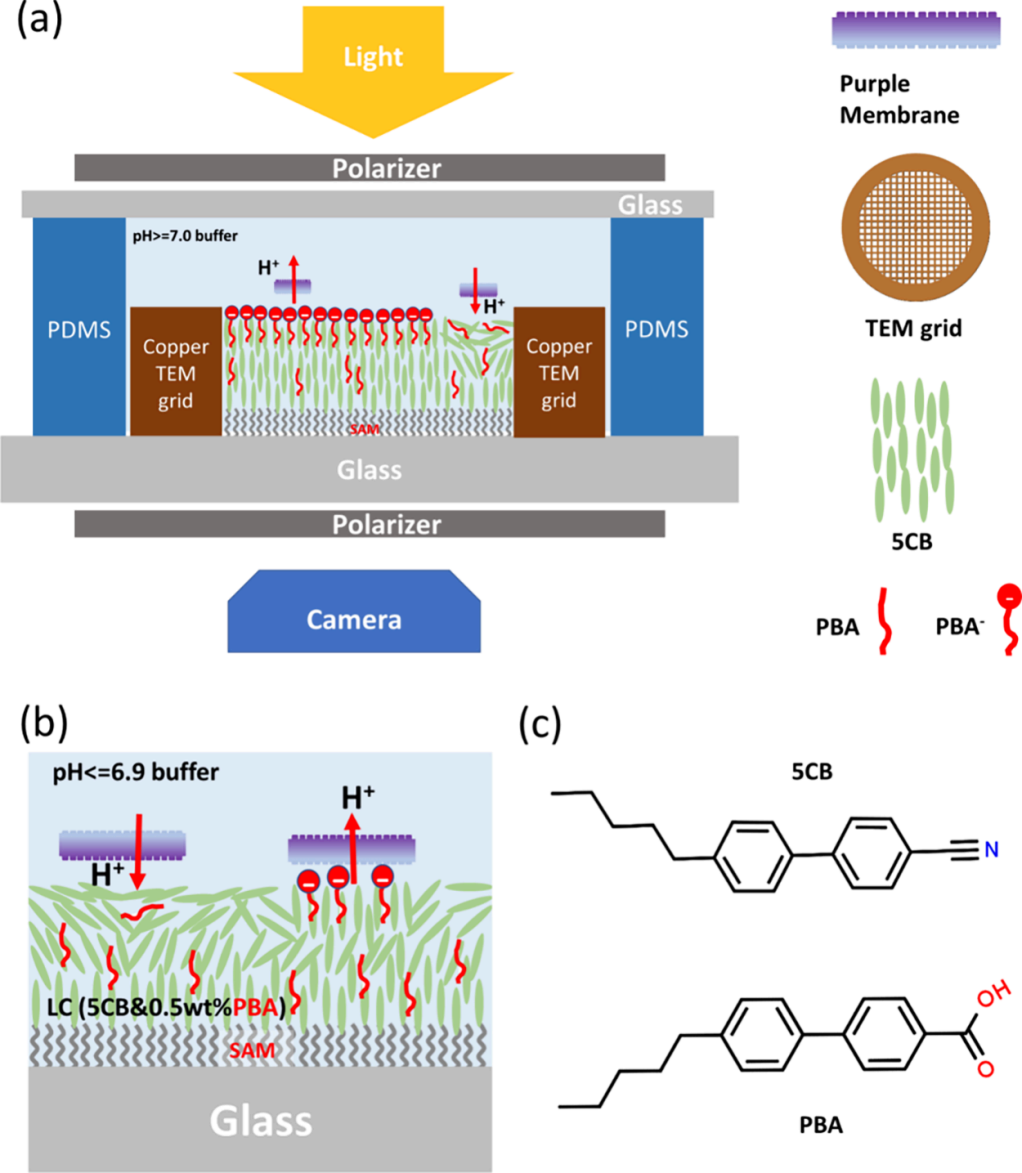
Schematics of the pH-responsive TEM-grid LC biosensor for the detection of the biofunction of PMs (a) in a pH ≥ 7 buffer and (b) in a pH ≤ 6.9 buffer. (c) Molecular structures of 5CB (4-cyano-4′-pentyl-biphenyl) and PBA (4′-pentyl-[1,1′-biphenyl]-4-carboxylic acid).

Using this strategy, we successfully demonstrate in this paper that PMs are stable on the surface of LC in a buffer solution and the biofunction of bRs as proton pumps can be detected by a pH-responsive LC thin film biosensor based on a glass substrate with a silane self-assembled monolayer (SAM), transmission electron microscopy (TEM) grid, and acid-doped 4-cyano-4′-pentyl-biphenyl (5CB), as shown in Figure 1.

## Experimental Details

### Materials

4-Cyano-4′-pentyl-biphenyl (5CB), 4′-pentyl-[1,1′-biphenyl]-4-carboxylic acid (PBA), PBS tablets, potassium chloride (KCl), and copper TEM grids were purchased from Sigma (Merck Life Science UK Limited). 1*H*,1*H*,2*H*,2*H*-Perfluorodecyltriethoxysilane was purchased from Fluoro Chem, Ltd., UK. A Dow Corning Sylgard 184 kit (PMDS kit) was purchased from Sil-Mid Ltd., UK. Purple membranes were purchased from Cube Biotech, Germany. A schematic depicting the close-packed bRs in a PM patch are shown in Supporting Information (SI) Figure S1.

#### Construction of the LC Biosensor Using a TEM Grid

The TEM-grid LC biosensor was constructed using a method following previous reports but with specific modifications.^26−28^ We used silane (1*H*,1*H*,2*H*,2*H*-perfluorodecyltriethoxysilane) to construct SAMs on glass slides. The glass slides were cleaned in piranha solution (containing 70% H_2_SO_4_ and 30% H_2_O_2_, v%) at 80 °C for 15 min and then rinsed thoroughly with Milli-Q water and dried under a stream of nitrogen gas. The cleaned glass slides together with 5 μL of 1*H*,1*H*,2*H*,2*H*-perfluorodecyltriethoxysilane were left in a desiccator under vacuum overnight. The quality of the SAM was tested using a contact angle measurement. The glass surfaces were hydrophobic with an average contact angle of 114.8° ± 1.4° (see SI Figure S2).

To make the TEM-grid LC biosensor, a copper TEM grid was placed on a SAM-coated glass slide followed by adding 0.2 μL of 0.5 wt % PBA-doped 5CB LC onto the TEM grid. The glass slide with the TEM grid and LC was then heated to 50 °C. The excess LC was removed by a glass capillary tube. After cooling down to RT, a PDMS well with a volume of ∼100 μL was constructed around the TEM grid. The buffer containing PM patches was added into the PDMS well and then sealed with a glass coverslip.

#### Optical Observation

The color change of the LC biosensors was monitored using a Trinocular Metallurgical Polarizing Microscope equipped with a pair of linear polarizers and an 18 MP USB 3.0 Color CMOS C-Mount Microscope Camera (Amscope Ltd., UK).

#### Atomic Force Microscope (AFM) Imaging

The AFM imaging of PMs on mica and on LC thin films was carried out in peak-force tapping mode using a Bruker multimode 8 AFM with a flow cell at 20 °C.

#### Contact Angle Measurement

The static contact angle of the glass slides with a silane SAM was measured using an FTA1000 Drop Shape Analysis System (First Ten Angstroms Inc., Canada).

## Experimental Results

### Creating a pH-Sensitive Sensor

**Figure 2:**
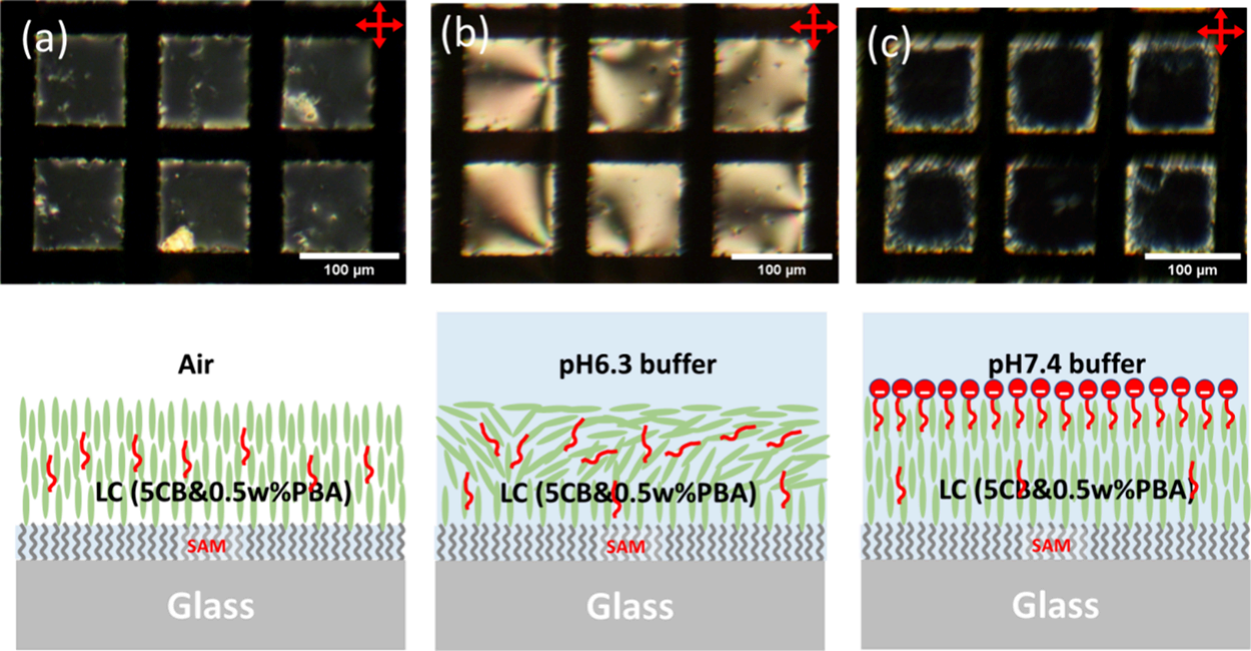
POM images of the TEM grid LC (5CB-0.5 wt % PBA) biosensor in transmission mode with crossed polarizers (a) in the air, (b) in pH 6.3 PBS buffer, and (c) in pH 7.4 PBS buffer.

The schematic in Figure 1 shows the essential features of the LC biosensor. The construction of the TEM grid LC biosensor is similar to that reported previously.^7^ In the air, the TEM-grid LC biosensor has a dark appearance under a polarized microscope with crossed polarizers, as shown in Figure 2a. This is because the LC molecules have a homeotropic alignment at both the LC–SAM and LC–air interfaces.^29^ Note that the SAM quality is important for the homeotropic alignment of LC at the LC–SAM interface. High-quality SAMs (water contact angle of ∼114°, SI Figure S2) give a reliable homeotropic alignment at the LC–SAM interface. In comparison, low-quality SAMs (water contact angle <90°) do not give a dark appearance under a polarized microscope, suggesting a tilted or homogeneous alignment of LC at the LC–SAM interface.^7^

A key aspect of our LC biosensor is the pH-responsive nature of the system. This attribute arises from the 0.5 wt % PBA doped in 5CB.^30^ PBA has a similar structure to 5CB but with a carboxylic acid functional group. At pH ≥ 7.0, PBA is deprotonated and has a negative charge. Attracted by the cations in the salt buffer, it can act as a surfactant and align the LC molecules at the LC-aqueous interface homeotropically. Therefore, the LC thin film, which is also homeotropically aligned at the glass–LC interface due to the SAM layer on the glass surface, will appear completely dark under a polarizing optical microscope (POM) with crossed polarizers. At pH ≤ 6.9, PBA is neutral and diffuses freely inside the LC thin film, having little effect on the surface alignment of LCs. Since water can align LC molecules homogeneously at the LC–water interface, the LC thin film should appear bright/colorful under the POM with crossed polarizers.

The behavior of our 5CB-PBA LC thin film biosensor system was first tested with buffers of different pH levels. When a pH 6.3 PBS buffer was added to the PDMS well, the LC biosensor was bright/colorful under the PLM with crossed polarizers, as shown in Figure 2b. As discussed above, this is because the LC crystal molecules have a homogeneous alignment at the top LC-aqueous interface and a homeotropic alignment at the bottom LC–SAM–glass interface. When the buffer was changed to pH 7.4 PBS buffer, the LC biosensor turned dark again under the PLM, as shown in Figure 2c, suggesting that the LC molecules are aligned homeotropically at the LC–water interface at pH 7.4. A pure 5CB TEM grid biosensor does not show any difference at these pH values, demonstrating that the LC biosensor is pH-responsive due to the existence of PBA in 5CB, as discussed above. The PBA molecule will be negatively charged at a pH higher than its isoelectric point; however, it should be noted that the isoelectric point of PBA at the LC-aqueous interface might differ from the value in bulk solution.^30,31^ Furthermore, we note that PBA doping by itself is not sufficient to make the LC biosensor pH-responsive. A suitable concentration of salt (cations) in the buffer is also required to attract deprotonated PBA to the surface of the LC thin film.

The pH response of the 5CB-PBA LC thin film biosensor was further studied in more detail over the pH range from 6.7 to 7.0, as shown in SI Figure S3. It was found that the LC biosensor showed an abrupt change between pH 6.9 and pH 7.0. It is bright and colorful at pH 6.9 or below; however, it is dark at pH 7.0 or above. The switching between these two states (bright and dark) at pH 6.9 and pH 7.0 was rapid and occurred within a few minutes after buffer exchange. This pH range of switching suggested that the PBA-doped LC biosensor is very sensitive to the proton concentration in the solution, which is advantageous for studying the biofunction of the purple membrane (PM) as a proton pump.

### Sensing pH Changes Induced by Purple Membranes

Purple membrane patches were first characterized using UV–vis spectroscopy and AFM. The absorbance spectrum of PM showed a peak at 570 nm, suggesting that PM was in its native state, as shown in SI Figure S4. The AFM images of PMs on mica clearly showed two orientations of PM patches: those with the cytoplasmic surface up (AFM measured thickness ∼8.7 nm), appearing as brighter patches in SI Figure S5a, and those with the extracellular surface up (AFM measured thickness ∼7.1 nm), appearing as darker patches in SI Figure S5a. The line profile of these patches is depicted in SI Figure S5b. The different thicknesses measured for the two orientations can be attributed to distinct interactions between PM patches and the mica substrate, as well as interactions between PM patches and the AFM tips.^32^ High-resolution AFM images of PM at the cytoplasmic surface and the extracellular surface show a hexagonal arrangement of bR trimers (SI Figure S5c,d) and are similar to previous reports.^33,34^ However, we note that imaging PMs directly on LC thin films in an aqueous solution did not yield clear AFM images of PMs since they are mobile under the AFM tip.

**Figure 3:**
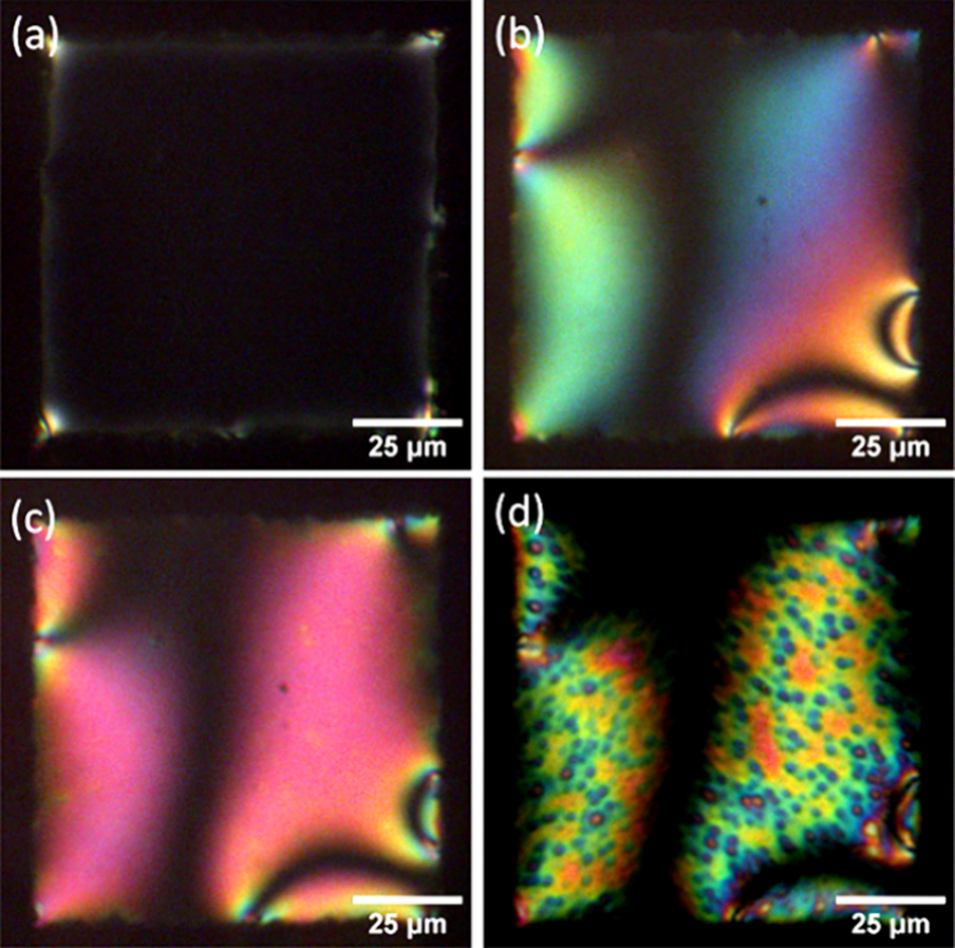
POM image of the TEM grid LC (5CB-0.5 wt % PBA) biosensor in transmission mode with crossed polarizers (a) in the air (without PM), (b) in pH 6.9 buffer (×100 times diluted PBS, 150 mM KCl) (without PM), (c) 15 min after adding PM (50 μg/mL) under ambient light, and (d) 20 min after POM observation with full-power light.

Having established that the LC biosensor is pH sensitive over the 6.7 to 7.0 range and that the PMs were in their native states, we investigated whether any local pH changes created by the light-driven proton pumping function of PMs could be observed. Before the addition of PMs, the biosensor image appeared dark in the air under the POM as expected, as shown in Figure 3a. When the buffer (×100 times diluted PBS, 150 mM KCl, pH6.9) was added into the PDMS well, the biosensor showed a colorful, bright pattern under the POM, suggesting a homogeneous alignment of LC molecules at the LC–water interface, as shown in Figure 3b. Due to the thickness variance of the 5CB thin film and the different in-plane alignment of LC molecules, the appearance of LC biosensors varies slightly from cell to cell, as shown in SI Figure S6. A ×100 times diluted PBS buffer was used because undiluted PBS buffer has a strong buffer capacity, which would reduce the sensitivity for observing the local pH change induced by PMs.

PM patches were then deposited onto the 5CB-PBA TEM by adding PM solution into the pH 6.9 buffer in the PDMS well (at 50 μg/mL final concentration). Due to the density difference between PMs and water, the PM patches sink to the bottom of the PDMS well in a few minutes and cover local regions at the surface of the LC thin film. After 15 min in darkness or ambient light, the appearance of the LC biosensor exhibited a minimal change under the POM. Only some very faint, light yellow spots appeared in the purple-colored background, as shown in Figure 3c, which are attributed to the existence of PMs at the LC–water interface. Although the light from the microscope lamp or ambient environment could power the PMs to transport a trace number of protons across the membrane, the effect is not obvious in POM images.

However, when the transmission lamp light of POM was switched on at its maximum level (power density ∼2 mW/cm^2^), the functioning of PMs was observed immediately, with a patchwork pattern of dark and light spots appearing within the colorful pattern of the LC thin film. Figure 3d shows the image of the biosensor under the POM after 20 min of exposure to the full-power lamp light from POM. In the pH 6.9 buffer, the LC thin film changes color when the pH value near the LC surface is increased to pH 7 or above. This suggests that the PM patches pumping protons upward across the PM will induce an obvious color change. The dark spots and patches became more obvious with time, as shown in SI Video S1, which suggests that a local pH change occurs in an accumulated manner. In the video, it can be seen that the dark spots are mobile and merge with other spots to form big patches. In some other samples, dark and bright spots were observed simultaneously, which suggests that the PM patches pumping protons in the opposite downward direction also induce a color change due to the lowering of local pH values (SI Figure S7 and SI Video S2).

The spots in Figure 3d were a good indication that PMs were functional—pumping protons across the membrane and generating a local pH change at the LC–PM interface, which will induce the local realignment of LC molecules in the LC thin film. A schematic explaining this result for pH 6.9 buffer is shown in Figure 1b.

**Figure 4:**
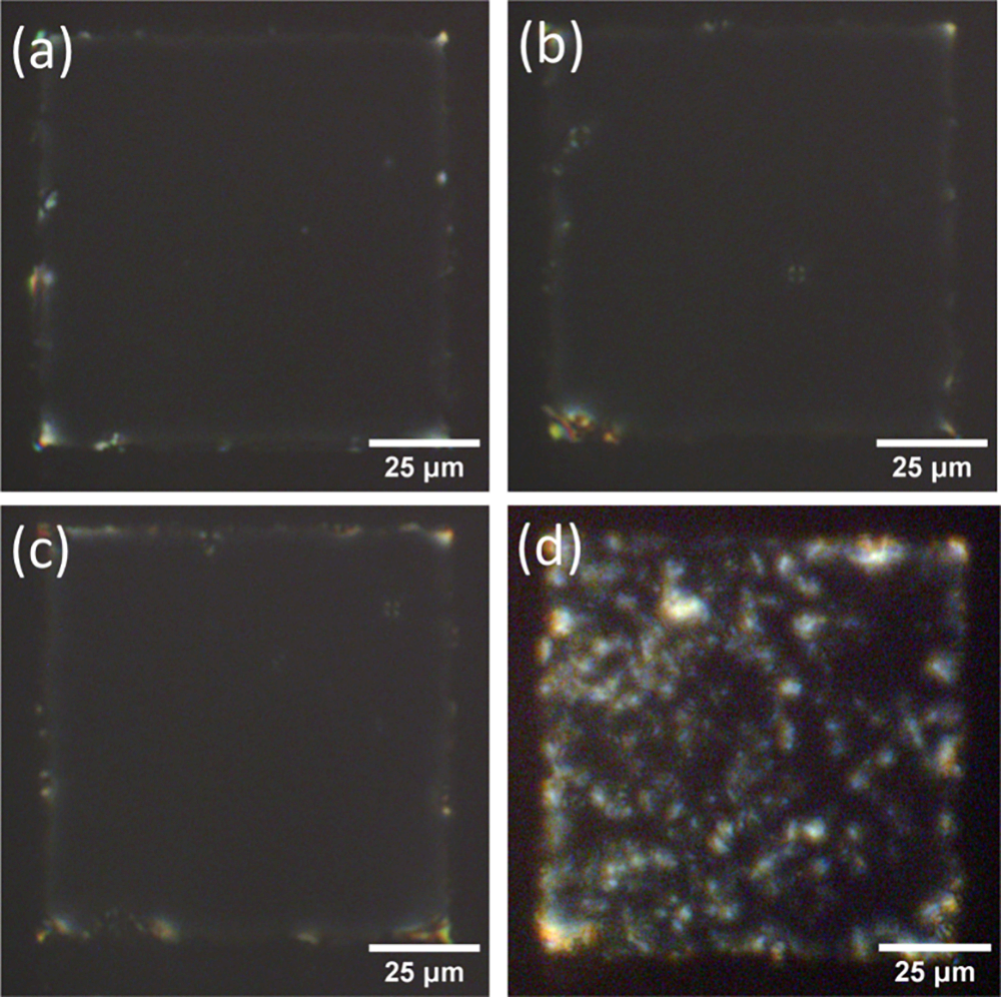
POM images of the TEM grid LC (5CB-0.5 wt % PBA) biosensor in transmission mode with crossed polarizers (a) in the air, (b) in pH 7.0 buffer (×100 times diluted PBS, 150 mM KCl), (c) 15 min after adding PM (50 μg/mL) under ambient light, and (d) 20 min after POM observation with full-power light.

To confirm that this color change of LC thin film is indeed induced by PMs, we carried out the same experiment in a pH 7 buffer to observe the operation of PM patches. The LC biosensor initially appeared dark in the air or in pH 7 buffer, as shown in Figure 4a,b, which is as expected—the alignment of LC molecules at the LC–air and LC–water interface is homeotropic, as shown in Figure 2. When PMs were added, the dark background was not changed, as shown in Figure 4c. However, after 20 min of full-power POM lamp light exposure, local bright spots are observed within the dark background, as shown in Figure 4d. This suggests that some PM patches pump protons downward across PM patches, which reduces the pH in the area beneath PMs, as shown schematically in Figure 1a. A decrease in pH would result in the homogeneous alignment of LC molecules at the interface, which results in a bright dot in the black background under the POM. SI Video S3 records the changes observed at 1 min intervals.

These results indicate that PMs exhibit two distinct orientations when deposited on the LC biosensor. This dual orientation results in PMs pumping protons in opposite directions, which can be observed in pH 6.9 and pH 7.0 buffers. We note that PMs will orient randomly on the LC biosensor if the interactions between both surfaces of the PMs and the LC thin film are similar. In such a scenario, each surface of the PMs would have an equal likelihood of facing upward. In practice, the interaction between the two surfaces of PM patches and the substrate may be influenced by various factors such as surface potential, pH, and salt concentration in the buffer. In our experiments, conducted in a buffer with a near-neutral pH and a weakly charged LC thin film surface, we observed that the PMs oriented in two directions with nearly equal probabilities, as illustrated in Figures 3 and 4.

### Control Experiments

**Figure 5:**
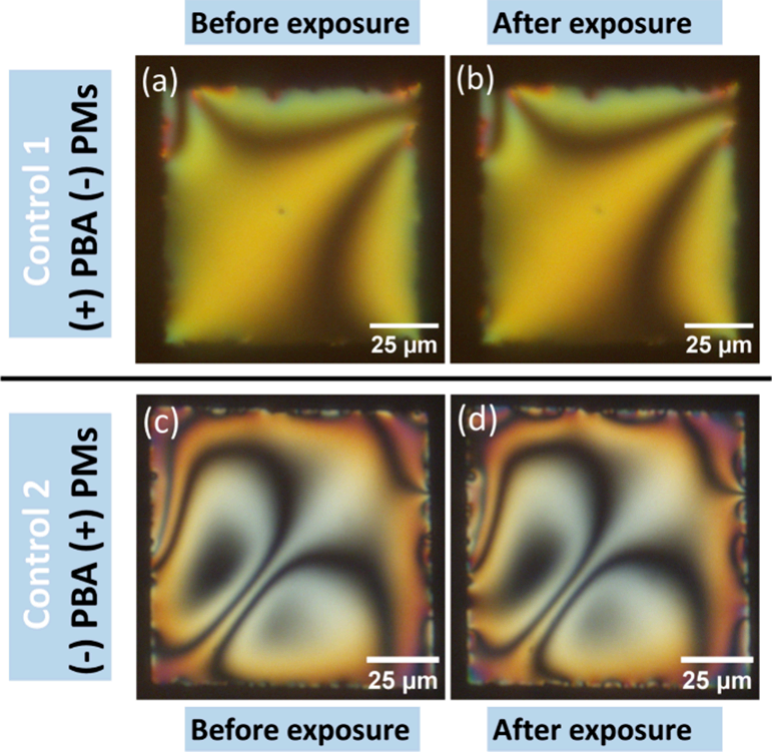
Control experiments: (a, b) with PBA in 5CB but without PMs on the surface of the LC thin film and (c, d) without PBA in 5CB but with PMs on the surface of the LC thin film. POM images of the TEM grid LC (5CB-0.5 wt % PBA) biosensor in transmission mode with crossed polarizers (a) in pH 6.9 buffer (×100 times diluted PBS, 150 mM KCl) and (b) 20 min after continuous POM observation with full-power lamp light without adding PMs. POM images of the TEM grid LC (5CB) biosensor in transmission mode with crossed polarizers (c) 15 min after adding PM (50 μg/mL) under ambient light in a pH 6.9 buffer (×100 times diluted PBS, 150 mM KCl) and (d) 20 min after continuous POM observation with full-power lamp light.

We carried out two control experiments to confirm that the change in the color of the LC thin film is caused by the pH-induced response of PBA-doped 5CB LC to the proton pumping function of the PM. In control experiment 1, the procedure depicted in Figure 3 was replicated, but without the inclusion of PMs. The 5CB-PBA thin film was observed to be bright and colorful when exposed to a pH 6.9 buffer. Without adding PMs, it was exposed to the full-power lamp light. No obvious change was observed in the 5CB-PBA thin film after 20 min of full-powered lamp light exposure, as shown in Figure 5a,b. We also carried out this control experiment in a pH 7.0 buffer. No obvious change was observed in 20 min without PMs, as shown in SI Figure S8. This suggests that the PM-induced local pH change (not the light) is indeed driving the color changes observed in Figures 3 and 4. Without PMs, the 5CB-PBA thin film did not change under light in the experimental time scale.

In control experiment 2, we repeated the experiment shown in Figure 3 but with just the pure 5CB LC. The 5CB thin film is dark in the air and bright in pH 6.9 or pH 7.4 buffer. However, no change was observed after the addition of PMs (Figure 5c), even after 20 min of full-powered lamp light exposure, as shown in Figure 5d. This result has two important implications: (1) the local pH change induced by PMs has no effect on the alignment of 5CB molecules as 5CB is not pH-responsive; (2) the lipids in the PM patches remain integrated with bRs and do not transfer to the surface of LC since even a trace amount of lipid has been reported to induce orientation changes in LC droplets or films,^35^ which is not observed here.

### Simplified LC Biosensor System

**Figure 6:**
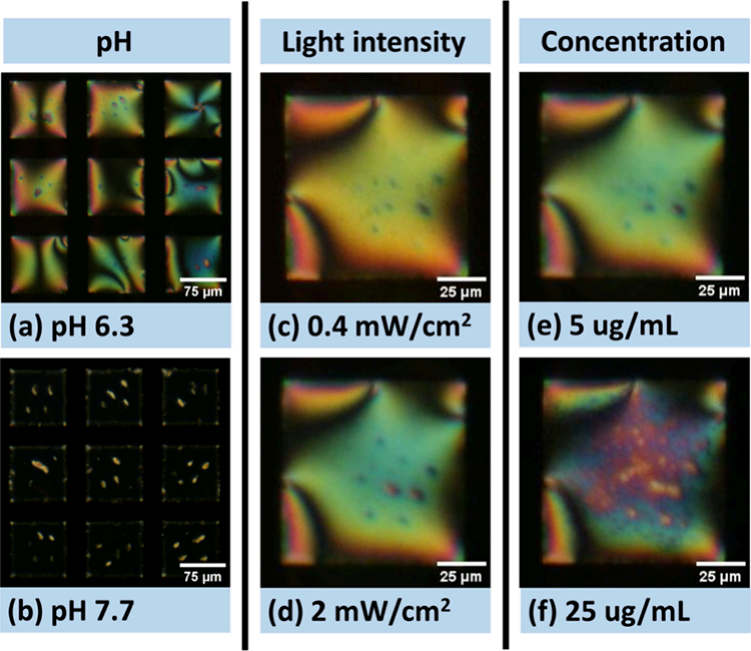
POM images of the TEM grid LC (UV-treated 5CB) biosensors (captured in transmission mode with crossed polarizers). (a, b) With 5 μg/mL PMs in different buffers (×10 times diluted PBS, 150 mM KCl) after 20 min of exposure to 2 mW/cm^2^ light. (c, d) With 5 μg/mL PMs in pH 6.3 buffers after 20 min of exposure to light with different intensities. Note: The experiment for (d) follows the experiment for (c). (e, f) Different concentrations of PMs in pH 6.3 buffers after 10 min exposure to 2 mW/cm^2^ light. Note: The experiment for (f) follows the experiment for (e) by adding additional 20 μg/mL PMs.

It is clear from the sections above that PBA doping of 5CB LC is essential for pH sensing. To simplify the LC sensor further, we investigated whether a UV-treated 5CB thin film biosensor^36^ would also be capable of monitoring the biofunction of PMs. Therefore, a (15 h, 365 nm UV, 100 μW/cm^2^) UV-treated PBA sensor was created following the general framework in Figure 1, and patches of PM were added. This UV-treated 5CB thin film biosensor has a transition between pH 7 and pH 7.7, as shown in SI Figure S9. When this system is illuminated with light, similar results were observed as those for 5CB-PBA thin film biosensors. In low pH buffers, new dark-colored dots appeared in the bright, colorful background, as shown in Figure 6a, while in high pH buffers, new bright/colorful dots appeared in the black background (Figure 6b).

Interestingly, the movement of PM patches on the LC thin film surface is clearly observed, especially at the lower concentrations of PMs (5 μg/mL), where the individual dots are very mobile. Furthermore, PM patches with the same orientations can aggregate, as indicated by the merging of colorful patterns in the POM images of LC thin films, as seen in SI Videos S4–S7.

The effect of different light intensities on the appearance of LC biosensors was also compared, as shown in Figure 6c,d. Stronger light intensity results in more obvious dots after 20 min of exposure to light, either due to the accumulation of pH change or due to the merging of small patches. Figure 6e,f shows the POM images of the UV-treated 5CB biosensors with different concentrations of PMs (5 and 25 μg/mL, respectively) after 10 min of full-power POM lamp light exposure in pH 6.3 buffer. More dots were observed for the biosensor with 25 μg/mL PMs. SI Videos S5–S8 show the change in the optical appearance of LC biosensors in different pH buffers and with different PM concentrations.

**Figure 7:**
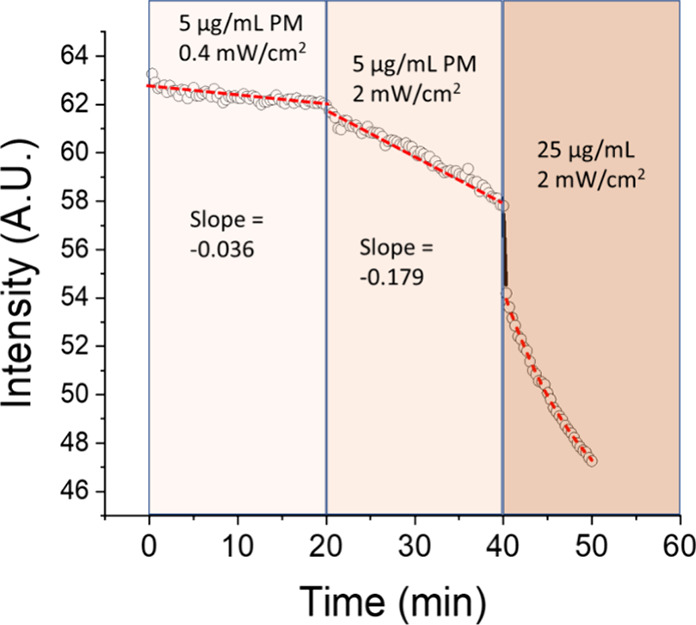
Change of the mean intensity of the POM images of the LC biosensor in SI Videos S5–S7 as a function of time and PM concentration and/or light intensity. At 20 min, the intensity of light was increased from 0.4 to 2 mW/cm^2^, and at 40 min, more PMs were added and the concentration of PMs was increased from 5 to 25 μg/mL. SI Video S4: initial PM concentration = 5 μg/mL, light intensity = 0.4 mW/cm^2^; SI Video S5: PM concentration retained at 5 μg/mL, increased light intensity = 2 mW/cm^2^; SI Video S6: PM concentration increased to 25 μg/mL, light intensity = 2 mW/cm^2^.

A detailed analysis of the change of mean intensity (the average brightness value across all pixels) of the images of the LC biosensor in SI Videos S4–S6 suggests a linear relationship between the intensity of images and light exposure time, especially for the low exposure intensity (0.4 mW/cm^2^) and low PM concentration (5 μg/mL), as shown in Figure 7. This general behavior was observed reproducibly over a number of experiments. This could be due to the linear increase in the numbers of transported protons or due to diffusion processes at the LC-aqueous interface near PMs. However, the changes are nonlinear at a higher exposure intensity and at a higher PM concentration (2 mW/cm^2^, 25 μg/mL) (SI Video S6), which may be due to the merging of the patches or the onset of the saturation of the OH^–^ concentration near PM patches.

## Discussion

Studying membrane proteins using LC biosensors is not an easy task. The challenges are in the integration of the lipid membrane, membrane protein, and LCs into one system. There have been some attempts to integrate lipid membranes with LCs previously; however, further integration of membrane protein, lipid membranes, and LCs has not been demonstrated.^21,22^ Our study bypassed this difficulty by using very stable purple membranes, which are 2D crystalline forms of bacteriorhodopsins embedded in their native lipids, which are found in photosynthetic bacteria such as *Halobacterium salinarum*.^24,37^ They can transport protons across the cell membranes under illumination to generate a proton gradient. Due to their stability, the biofunction of purple membranes as a proton pump has been demonstrated in various optoelectronic device applications, such as photovoltaics and sensors.^38,39^

In this study, we combined LC biosensors and PMs enabling the proton gradient across PMs to be detected and converted to optical signals by a pH-sensitive LC biosensor. Due to the density difference between PMs and water, PMs readily sank to the surface of the LC thin film with no need for surface modification of LCs. The transfer of lipids from PM to LC was not observed in our experiments, with the PMs remaining in their native state as shown by the local pH changes detected by our LC biosensors, which is the main result of the paper. As far as we know, this is the first LC biosensor demonstrated for transmembrane proteins.

The μm-scale local pH changes (below or around PM patches) were directly observed from the change of the POM images of LC thin films, as shown in Figures 3 and 4. Due to the different orientations of PM patches, the local pH changes are bidirectional, which gives different colored patches in the POM images of LC thin films, especially in low pH buffers.

The other novelty of this study is that we have used a special pH-sensitive LC biosensor to monitor the local pH change introduced by PMs. This is a rare example of an LC thin film biosensor that has been used to monitor the local environment changes at μm length scales, though two kinds of pH-sensitive LC biosensors have been reported previously for the detection of various enzyme activities. For example, poly(acrylicacid-*b*-4-cynobiphenyl-4-oxyundecyl acrylate)-coated 5CB LC droplets have been demonstrated for the detection of glucose with glucose oxidase immobilized to the PAA chains.^40^ This type of LC biosensor needs a specially synthesized polymer and the linkage of glucose oxidase to polymer chains.^41^ Another type of pH-sensitive LC biosensor relies on doping pH-sensitive molecules in the LC.^42^ For example, PBA-doped 5CB and stearic acid-doped 5CB have been used for the detection of penicillin and urease.^30,43^ UV-treated 5CB, which contains 4-cyano-4′-biphenylcarboxylic acid, has been used for the detection of xanthine, glucose, cholesterol, and urease.^36,44−46^ These kinds of biosensors are mainly used in the form of LC thin films or LC droplets immobilized on the surface.^43^ The doped acidic molecules work as amphophile molecules/surfactants at high pH when the carboxylic acid group is deprotonated (COOH to COO^–^), therefore aligning 5CB molecules at the LC-aqueous interface vertically to the surface. Compared to pH-responsive LC biosensors of the first kind, these LC biosensors are much easier to fabricate and more sensitive. However, both kinds of LC biosensors were used to monitor pH changes at a large scale, not at a microscale. Our study suggests that these biosensors are also useful for monitoring local environmental changes. However, their full potential has not been fully explored and they may find wider applications in biological research. For example, pH-responsive LC biosensors could be used for the detection of environmental change induced by bacteria or mammalian cells.^47^ More recently, the investigation on pH-responsive cholesteric LC biosensors has also emerged.^48^

We want to note that in this study, the pH changes induced by PMs were monitored qualitatively. Establishing a correlation between these color changes in the LC thin film and actual pH values would be very interesting but presents significant challenges. To achieve this, the use of pH-sensitive dyes as local calibration tools could be explored, which requires further investigation.

Though in this study, the biofunction of BRs is studied in a qualitative rather than a quantitative way, our study provides a platform for further studies on the biofunction of membrane proteins using LC biosensors. For example, this pH-sensitive biosensor is also sensitive to salt concentration; therefore, it has the potential to be used for the study of membrane proteins that pump sodium or calcium ions.^49^

## Conclusions

In this work, we successfully constructed a pH-responsive TEM-grid LC (PBA-doped 5CB) thin film biosensor for the detection of the biofunction of purple membranes as a proton pump. This LC biosensor appears bright and colorful in pH ≤ 6.9 buffers and dark in pH ≥ 7 buffers. An abrupt switch between these two states was observed around pH 6.9–7.0. PMs were positioned in a stable manner on the LC surface and no noticeable transfer of lipids from PMs to the LC surface was observed by POM.

With POM lamp light illumination, the functioning of PMs was revealed by the optical appearance change of the LC biosensor under the POM with crossed polarizers. In the pH 6.9 buffer, many new-born bluish dots appeared in the colorful/bright image of the LC thin film. In the pH 7.0 buffer, bright dots appeared in the dark image of the LC thin film. Control experiments showed that the color changes were indeed due to the pH-responsive property of PBA and the local pH change caused by PMs at the LC–PMs interface.

This kind of biosensor was not only pH-sensitive but also salt-sensitive. Therefore, it can also be used for the detection of the local concentration change of other cations, such as Na^+^ or Ca^2+^, which are important for many biological activities. This research establishes the potential for studying important biofunctions of membrane proteins, especially ion-channel proteins using LC biosensors.

## Supporting Information Available

Video S1: The POM (transmission mode, crossed polarized) images show the change in the appearance of a 5CB-PBA (0.5 wt%) thin film biosensor in pH 6.9 (×100 times diluted PBS, 150 mM KCl) buffer with 50 μg/mL PMs under full-power lamp light (2 mW/cm^2^) at 3 frames/minute (after 20 min exposure to 1mW/cm^2^lamp light) (AVI)
Video S2: The POM (transmission mode, crossed polarized) images show the change in the appearance of a UV-treated 5CB thin film biosensor in pH 6.4 (×10 times diluted PBS, 150 mM KCl) buffer with 5 μg/mL PMs under full-power lamp light (2 mW/cm^2^) at 2 frames/minute (AVI)
Video S3: The POM (transmission mode, crossed polarized) images show the change in the appearance of a 5CB-PBA (0.5 wt%) thin film biosensor in pH 7.0 (×100 times diluted PBS, 150 mM KCl) buffer with 50 μg/mL PMs under full-power lamp light (2 mW/cm^2^) (3 frames/minute) (AVI)
Video S4: The POM (transmission mode, crossed polarized) images show the change in the appearance of a UV-treated 5CB thin film biosensor in pH 6.3 (×10 times diluted PBS, 150 mM KCl) buffer with 5 μg/mL PMs under 20% of full-power lamp light (∼0.4 mW/cm^2^) for 20 min at 3 frames/minute (AVI)
Video S5: The POM (transmission mode, crossed polarized) images show the change in the appearance of a UV-treated 5CB thin film biosensor in pH 6.3 (×10 times diluted PBS, 150 mM KCl) buffer with 5 μg/mL PMs under full-power lamp light (2 mW/cm^2^) for 20 min at 3 frames/minute (note: Video S6 is following S5) (AVI)
Video S6: The POM (transmission mode, crossed polarized) images show the change in the appearance of a UV-treated 5CB thin film biosensor in pH 6.3 (×10 times diluted PBS, 150 mM KCl) buffer with 25 μg/mL PMs under full-power lamp light (∼2 mW/cm^2^) at 3 frames/minute (AVI)
Video S7: The POM (transmission mode, crossed polarized) images show the change in the appearance of a UV-treated 5CB thin film biosensor in pH 7.7 (×10 times diluted PBS, 150 mM KCl) buffer with 25 μg/mL PMs under full-power lamp light (∼2 mW/cm^2^) at 3 frames/minute (AVI)
Diagrams illustrating PMs, contact angle measurements on SAM-coated glass, pH responses of TEM grid LC biosensors using PBA, UV–vis spectra of PMs, AFM images of PMs on mica in PBS, POM image of the LC biosensor in pH 6.9 buffer, time-sequence POM images of LC biosensor with PMs in action in pH 6.4 buffer, control experiment in pH 7.0 buffer, and pH responses of LC biosensors using UV-treated 5CB (PDF)

## Supplementary Material

am4c06614_si_001.avi

am4c06614_si_002.avi

am4c06614_si_003.avi

am4c06614_si_004.avi

am4c06614_si_005.avi

am4c06614_si_006.avi

am4c06614_si_007.avi
