# Supplementary material for: Membrane Proteins in Action Monitored by pH-Responsive Liquid Crystal Biosensors
Source: ACS Appl Mater Interfaces. 2024 Jun 6;16(24):31843–50. doi: 10.1021/acsami.4c06614 (PMC11194810; doi:10.1021/acsami.4c06614)
Supplement: Supplementary file 8 — am4c06614_si_008.pdf [file am4c06614_si_008.pdf]

Supporting information: Membrane proteins in action monitored by pH-responsive liquid crystal biosensors

Peng Bao, Kyle Phillips, Rasmita Raval*

Open Innovation Hub for Antimicrobial Surfaces, Surface Science Research Centre, Department of Chemistry, University of Liverpool, Liverpool L69 3BX, UK

Corresponding author: Dr Peng Bao, e-mail: peng.bao@gmail.com; Prof. Rasmita Raval, e-mail: raval@liverpool.ac.uk

Supporting information figures:

Figure S1. (a) *Halobacterium salinarum* cell with patches of PM. (b) BR trimers (Seven transmembrane α-helices assembled into trimers) (c) Schematics of PM patch embedded with bR trimers packed in a 2D hexagonal crystalline lattice. [ref S1]

Figure S2. The contact angle measurement of the silane SAM-coated glass suggests that a high-quality of SAM layer has been formed on the glass surface (with a contact angle of 114°). (Instrument used: FTA-1000, First Ten Angstroms, USA)

Figure S3 The POM image of the TEM grid LC (5CB-0.5 wt% PBA) biosensor in transmission mode with crossed polarizers in PBS buffers at different pH (a) pH6.7; (b) pH6.8; (c) pH6.9; (d) pH7.0.

Figure S4. The absorbance spectrum of PMs in water (20 µg/mL) shows an absorbance peak at 570nm. (Instrument used: Shimadzu UV-1800 spectrophotometer).

Figure S5. The AFM image of purple membrane on mica in PBS buffer (pH 7.4). (a) 2um scan; (b) height profile analysis suggests the two orientations of PM on mica show two different heights at ~ 7.1 nm (extracellular surface up) and ~ 8.7 nm (cytoplasmic surface up) respectively. (c)a high-resolution image of the extracellular surface of PM; (d) a high-resolution image of the cytoplasmic surface of PMs. (Note: The AFM images were taken in the peak-force tapping mode using a Bruker multimode 8 AFM, the tip used is SNL10-A).

Figure S6 The POM image of the TEM grid LC (5CB-0.5 wt% PBA) biosensor in pH6.9 buffer (x100 times diluted PBS, 150 mM KCl) taken in transmission mode with crossed polarizers.

Figure S7 The POM image of the TEM grid LC (UV treated 5CB) biosensor in pH6.4 buffer (x10 times diluted PBS, 150 mM KCl) taken in transmission mode with crossed polarizers under full-power lamp light exposure. Images were taken at different time points: (a) 2.5; (b) 5; (c) 7.5; (d) 10; (e) 12.5; (f) 15; (g) 17.5; (h) 20 minutes.

Figure S8 Control experiments: (a-b) with PBA in 5CB but without PMs on the surface of LC thin film; (c-d) without PBA in 5CB but with PMs on the surface of LC thin film. (a-b) The POM image of the TEM grid LC (5CB-0.5 wt% PBA) biosensor in transmission mode with crossed polarizers (a) in pH7.0 buffer (x100 times diluted PBS, 150 mM KCl); (b) 20 min after continuous POM observation with full-power lamp light without adding PMs. (c-d) The POM image of the TEM grid LC (5CB) biosensor in transmission mode with crossed polarizers (c) 15 min after adding PM (50 µg/mL) under ambient light in a pH7.0 buffer (x100 times diluted PBS, 150 mM KCl); (d) 20 min after continuous POM observation with full-power lamp light.

Figure S9 The POM image of the TEM grid LC (15 hr UV-treated 5CB) biosensor in transmission mode with crossed polarizers in the air and in different pH (x10 diluted PBS, 150 mM KCl) buffers. (a) In the air, (b) pH8.5; (c) pH7.7; (d) pH7; (e) pH6.7; (f) pH6.3.

Supporting information videos:

Video S1: The POM (transmission mode, crossed polarized) images show the change in the appearance of a 5CB-PBA (0.5 wt%) thin film biosensor in pH 6.9 (x100 times diluted PBS, 150 mM KCl) buffer with 50 µg/mL PMs under full-power lamp light (2 mW/cm^2) at 3 frames/minute (after 20 min exposure to 1mW/cm^2 lamp light).

Video S2: The POM (transmission mode, crossed polarized) images show the change in the appearance of a UV-treated 5CB thin film biosensor in pH 6.4 (x10 times diluted PBS, 150 mM KCl) buffer with 5 µg/mL PMs under full-power lamp light (2 mW/cm^2) at 2 frames/minute.

Video S3: The POM (transmission mode, crossed polarized) images show the change in the appearance of a 5CB-PBA (0.5 wt%) thin film biosensor in pH 7.0 (x100 times diluted PBS, 150 mM KCl) buffer with 50 µg/mL PMs under full-power lamp light (2 mW/cm^2). (3 frames/minute)

Video S4: The POM (transmission mode, crossed polarized) images show the change in the appearance of a UV-treated 5CB thin film biosensor in pH 6.3 (x10 times diluted PBS, 150 mM KCl) buffer with 5 µg/mL PMs under 20% of full-power lamp light (~0.4 mW/cm^2) for 20 min at 3 frames/minute.

Video S5: The POM (transmission mode, crossed polarized) images show the change in the appearance of a UV-treated 5CB thin film biosensor in pH 6.3 (x10 times diluted PBS, 150 mM KCl) buffer with 5 µg/mL PMs under full-power lamp light (2 mW/cm^2) for 20 min at 3 frames/minute. Note: Video S6 is following S5.

Video S6: The POM (transmission mode, crossed polarized) images show the change in the appearance of a UV-treated 5CB thin film biosensor in pH 6.3 (x10 times diluted PBS, 150 mM KCl) buffer with 25 µg/mL PMs under full-power lamp light (~ 2 mW/cm^2) at 3 frames/minute.

Video S7: The POM (transmission mode, crossed polarized) images show the change in the appearance of a UV-treated 5CB thin film biosensor in pH 7.7 (x10 times diluted PBS, 150 mM KCl) buffer with 25 µg/mL PMs under full-power lamp light (~ 2 mW/cm^2) at 3 frames/minute.

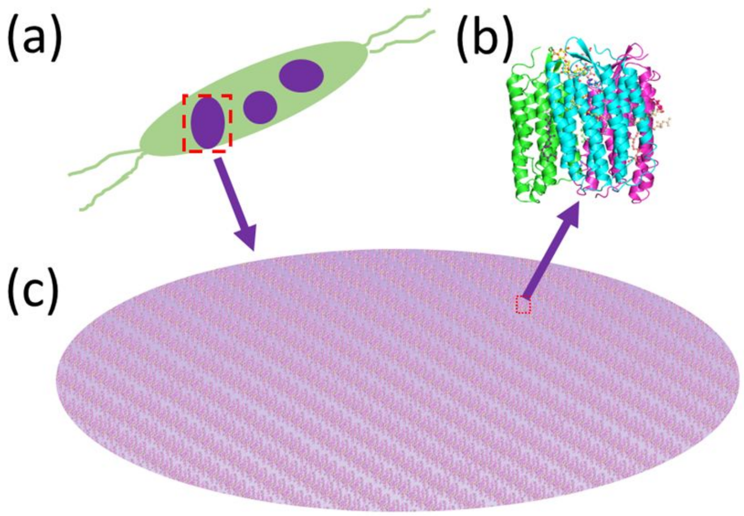

Figure S1. (a) *Halobacterium salinarum* cell with patches of PM. (b) BR trimers (Seven transmembrane α-helices assembled into trimers) (c) Schematics of PM patch embedded with bR trimers packed in a 2D hexagonal crystalline lattice. [ref S1]

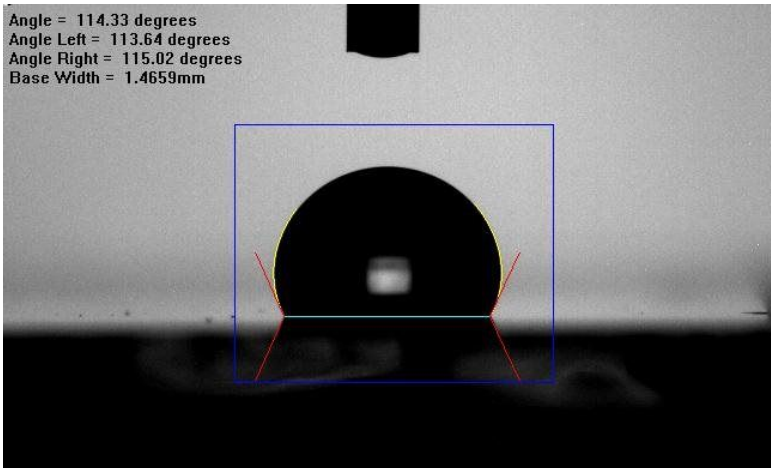

Figure S2. The contact angle measurement of the silane SAM-coated glass slides suggests that a high-quality SAM layer has been formed on the glass surface (with a contact angle of 114°). Before the coating of SAM, the contact angle of the cleaned glass slides was too low to be accurately measured. (Instrument used: FTA-1000, First Ten Angstroms, USA)

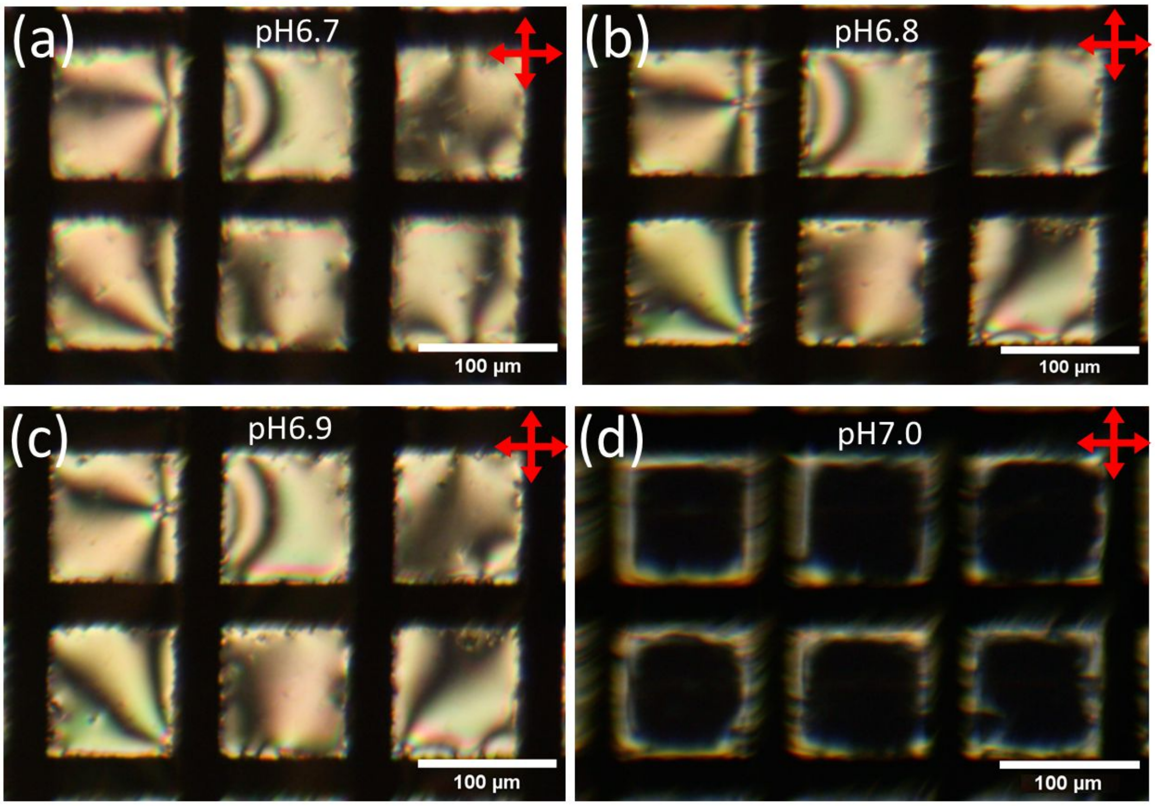

Figure S3 The POM image of the TEM grid LC (5CB-0.5 wt% PBA) biosensor in transmission mode with crossed polarizers in PBS buffers at different pH (a) pH6.7; (b) pH6.8; (c) pH6.9; (d) pH7.0.

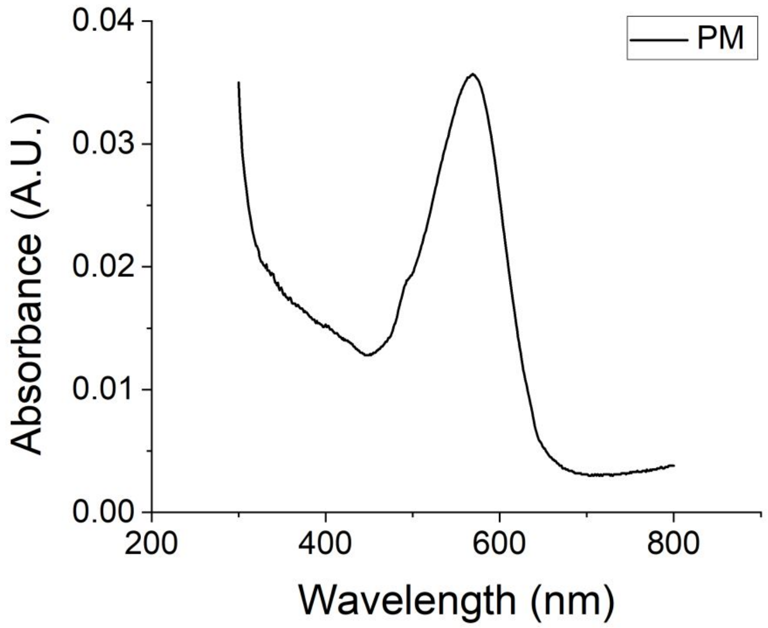

Figure S4. The absorbance spectrum of PMs in water (20 µg/mL) shows an absorbance peak at 570nm. (Instrument used: Shimadzu UV-1800 spectrophotometer)

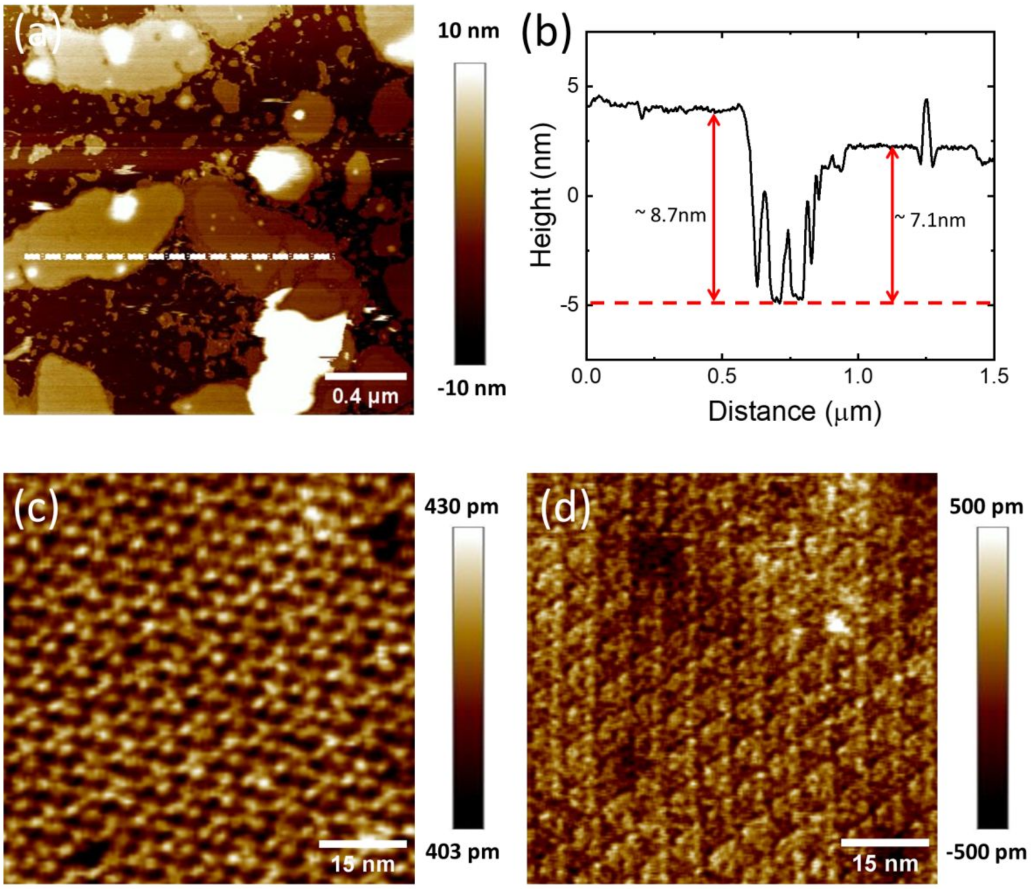

Figure S5. The AFM image of purple membrane on mica in PBS buffer (pH 7.4). (a) 2um scan; (b) height profile analysis suggests the two orientations of PM on mica show two different heights at ~ 7.1 nm (extracellular surface up) and ~ 8.7 nm (cytoplasmic surface up) respectively. (c)a high-resolution image of the extracellular surface of PM; (d) a high-resolution image of the cytoplasmic surface of PMs. (Note: The AFM images were taken in the peak-force tapping mode using a Bruker multimode 8 AFM, the tip used is SNL10-A).

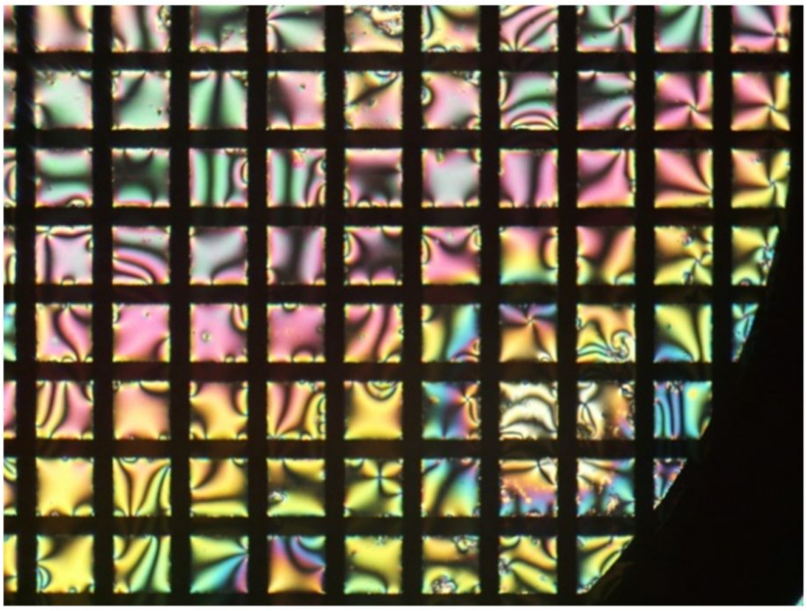

Figure S6 The POM image of the TEM grid LC (5CB-0.5 wt% PBA) biosensor in pH6.9 buffer (x100 times diluted PBS, 150 mM KCl) taken in transmission mode with crossed polarizers.

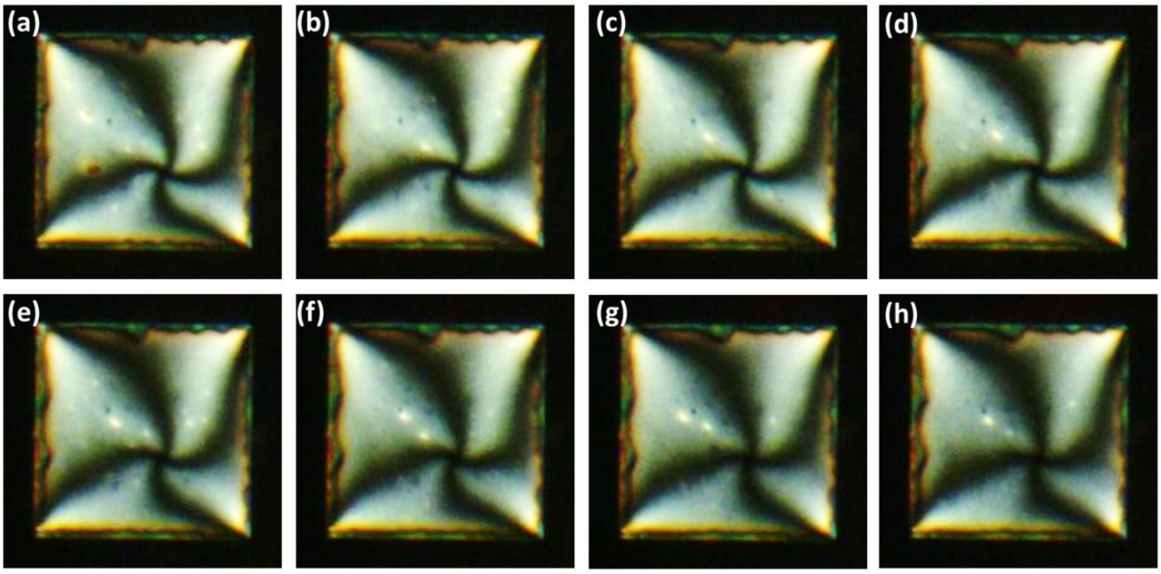

Figure S7 The POM image of the TEM grid LC (UV treated 5CB) biosensor in pH6.4 buffer (x10 times diluted PBS, 150 mM KCl) taken in transmission mode with crossed polarizers under full-power lamp light exposure. Images were taken at different time points: (a) 2.5; (b) 5; (c) 7.5; (d) 10; (e) 12.5; (f) 15; (g) 17.5; (h) 20 minutes.

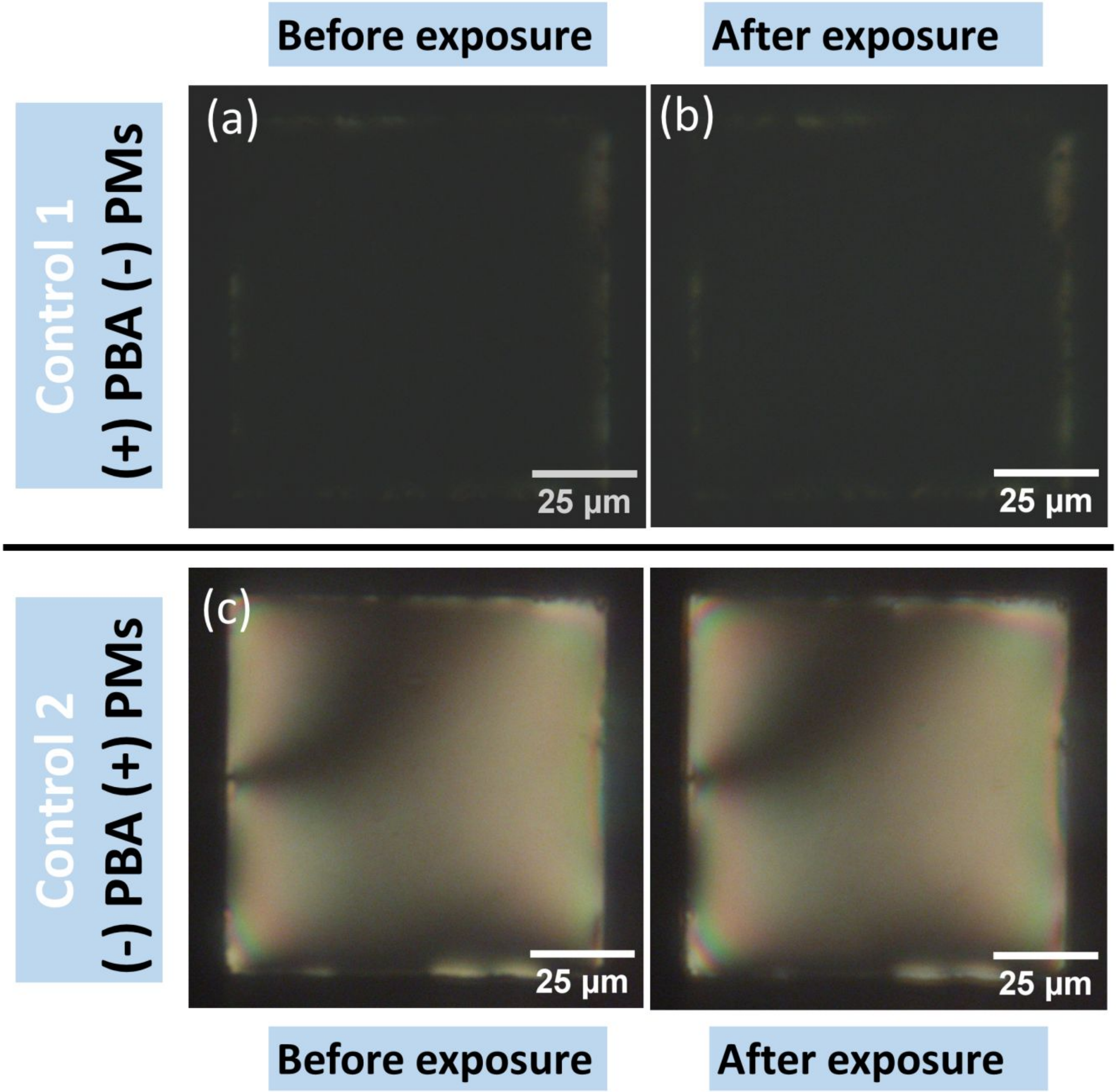

Figure S8 Control experiments: (a-b) with PBA in 5CB but without PMs on the surface of LC thin film; (c-d) without PBA in 5CB but with PMs on the surface of LC thin film. (a-b) The POM image of the TEM grid LC (5CB-0.5 wt% PBA) biosensor in transmission mode with crossed polarizers (a) in pH7.0 buffer (x100 times diluted PBS, 150 mM KCl); (b) 20 min after continuous POM observation with full-power lamp light without adding PMs. (c-d) The POM image of the TEM grid LC (5CB) biosensor in transmission mode with crossed polarizers (c) 15 min after adding PM (50 µg/mL) under ambient light in a pH7.0 buffer (x100 times diluted PBS, 150 mM KCl); (d) 20 min after continuous POM observation with full-power lamp light.

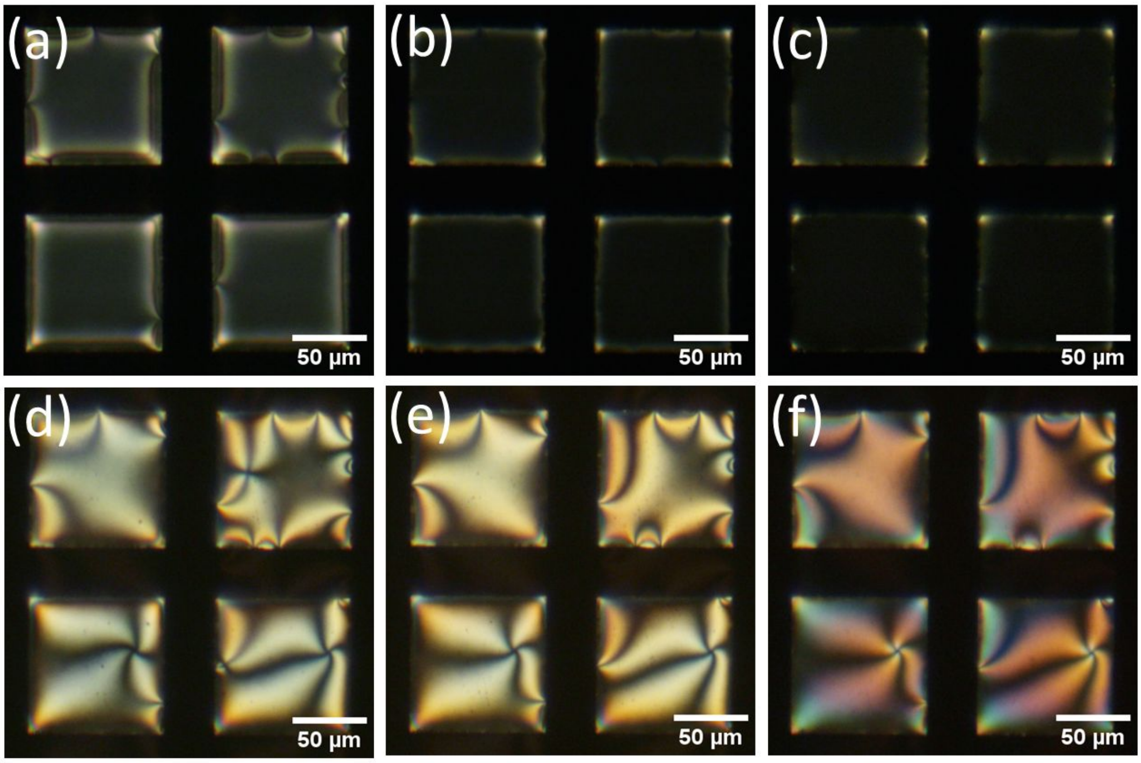

Figure S9 The POM image of the TEM grid LC (15 hr UV-treated 5CB) biosensor in transmission mode with crossed polarizers in the air and in different pH (x10 diluted PBS, 150 mM KCl) buffers. (a) In the air, (b) pH8.5; (c) pH7.7; (d) pH7; (e) pH6.7; (f) pH6.3.

References:

S1: de Q. Silveira, G., et al., *Energy Transfer Induced by Dye Encapsulation in a Hybrid Nanoparticle-Purple Membrane Reversible Assembly.* Advanced Functional Materials, 2019. **29**(43): p. 1904899.
